# Comparative Changes in Fecal Microbiome After Endoscopic Resection and Surgical Resection in Gastric Cancer Patients

**DOI:** 10.3390/jpm15040144

**Published:** 2025-04-04

**Authors:** Hochan Seo, Jae Yong Park, Hee Sang You, Beom Jin Kim, Jae Gyu Kim

**Affiliations:** 1 Laboratory of Gastrointestinal Mucosal Immunology, Chung-Ang University College of Medicine, Seoul 06974, Republic of Korea; d5394@cauhs.or.kr (H.S.); d5388@cauhs.or.kr (H.S.Y.); 2 Department of Internal Medicine, Chung-Ang University College of Medicine, Seoul 06974, Republic of Korea; jay0park@cau.ac.kr (J.Y.P.); kimbj@cau.ac.kr (B.J.K.)

**Keywords:** gastric cancer, gastrectomy, endoscopic submucosal dissection, gut microbiome

## Abstract

**Background/Objectives**: Gastric cancer treatments can lead to significant alterations to patients’ gastrointestinal microbiome. However, differences in microbial impacts between gastrectomy and endoscopic submucosal dissection (ESD) remain underexplored. This study investigates how these treatments influence microbial diversity and composition in patients with stage I gastric cancer. **Methods**: Patients with pathologically confirmed stage I gastric cancer were recruited from Chung-Ang University Hospital between December 2016 and December 2019. This study analyzed fecal samples from 13 patients (ESD: *n* = 5; gastrectomy: *n* = 8) before and after treatment using 16S rRNA gene sequencing. Microbial diversity indices and taxonomic composition were compared, with follow-up extending up to two years. **Results**: In the total cohort, alpha diversity significantly decreased post-treatment (*p* < 0.05), and beta diversity analysis showed distinct clustering between pre- and post-treatment samples (*p* < 0.05). At the genus level, Bacteroides significantly decreased (*p* < 0.05), while Lactobacillus, Bifidobacterium, and Blautia showed significant increases (*p* < 0.05). Comparative analyses revealed that in the ESD group, alpha diversity remained unchanged, although beta diversity showed significant clustering (*p* < 0.05), without notable changes in major taxa. In contrast, surgical resection resulted in a significant reduction in alpha diversity (*p* < 0.05) and beta diversity clustering (*p* < 0.05), with increased abundances of Streptococcus and Blautia and decreased abundance of Bacteroides (*p* < 0.05). **Conclusions**: Surgical gastrectomy exerts significant effects on microbiome diversity and composition, while ESD has a more limited impact. These findings underscore the importance of considering microbiome changes in postoperative management.

## 1. Introduction

Gastric cancer is one of the most prevalent malignancies worldwide, particularly in East Asia, where its incidence remains notably high [1]. The etiology of gastric cancer is multifactorial and involves genetic predisposition, environmental factors, and dietary habits. Helicobacter pylori infection is a well-established risk factor for the development of gastric cancer [2]. In addition to H. pylori, recent studies suggest that the broader gastrointestinal (GI) microbiome may also contribute to the progression of gastric cancer [3]. The GI microbiome plays a substantial role in maintaining digestive health and alterations in its composition and balance are associated with various GI diseases, including cancer [4].

The treatment of gastric cancer is guided by the tumor’s location, size, and stage. Endoscopic submucosal dissection (ESD) is a minimally invasive option for early-stage gastric cancer with negligible lymph node involvement, while gastrectomy is more commonly employed for advanced stages [5]. ESD offers oncological outcomes comparable to those of surgery, with the added advantage of preserving more of the stomach’s functional reserve and causing fewer structural alterations to the GI tract [6,7]. In contrast, gastrectomy involves considerable anatomical changes, including reduced acid secretion, altered bile acid metabolism, and altered food transit time, which can lead to notable changes in the gut microbiome [8].

Patients undergoing gastrectomy for gastric cancer often experience various GI symptoms after surgery, such as bloating, flatulence, abdominal discomfort, and diarrhea [9]. These symptoms can considerably affect the quality of life and recovery process. One proposed factor contributing to these symptoms is the alteration of the gut microbiome following surgery. Changes in microbial composition and diversity after gastrectomy are considered to affect gut function, potentially exacerbating or triggering GI symptoms [8]. Although the exact mechanisms remain unclear, there is a growing interest in understanding how shifts in the gut microbiome might play a role in post-gastrectomy digestive issues.

Recent studies have explored how the composition of the GI tract microbiome changes before and after surgical gastrectomy. However, existing studies on postsurgical microbiome changes have largely focused on bariatric procedures, with relatively few studies investigating the effects of surgery on patients with gastric cancer [10,11]. Most studies on gastric cancer compare patients with healthy controls, leaving a gap in understanding how microbiome changes differ between treatments, such as ESD and gastrectomy. Direct comparisons between these interventions are scarce, highlighting the need for research exploring the effects of different treatment modalities on the gut microbiome and patient recovery.

The aim of this study was to compare changes in the fecal microbiome of patients with gastric cancer following ESD and gastrectomy. By examining these differences, we sought to identify key microbial alterations after treatment and assess the influence of structural changes in the GI tract on the gut microbiome.

## 2. Materials and Methods

### 2.1. Study Participants and Sample Collection

This study utilized samples from a subset of patients enrolled in a previous study at Chung-Ang University Hospital (Seoul, Republic of Korea), where patients with gastric cancer were prospectively recruited from September 2017 to December 2019 and followed up for up to two years (6 months, 1 year, and 2 years) for metagenomic analyses [12]. In the previous study, patients newly diagnosed with gastric cancer were included. Patients with a history of gastric cancer or other malignancies for which they had undergone surgery, chemotherapy, or radiotherapy were excluded. Additionally, individuals diagnosed with gastric dysplasia, pregnant women, and those with a history of antibiotic or probiotic use within three months prior to enrollment were excluded.

In the current study, we included only patients diagnosed with stage I gastric cancer based on the final pathological evaluation according to the AJCC 8th edition criteria, to enhance comparability between the ESD and surgery groups [13]. Patients with a final pathology indicating stage II or higher gastric cancer and those without samples obtained from post-treatment follow-up were excluded from the analysis. This study was approved by the Institutional Review Board of the Chung-Ang University Hospital (IRB No. 1772-001-290). All methods used in this study were conducted in accordance with the Declaration of Helsinki, and informed consent was obtained from all participants.

Five patients who underwent ESD and eight who underwent distal gastrectomy were selected. Fecal samples were collected from patients prior to treatment and between 6 months and 2 years post-treatment. To ensure the integrity of the microbiome data, the patients were instructed to maintain their usual dietary habits and avoid alcohol consumption and overeating before sample collection. Additionally, they were advised not to use antibiotics or proton pump inhibitors (PPIs) within three months before stool collection to prevent any potential alterations in the microbiome. Patients in the ESD group received PPI therapy for 4–8 weeks post-procedure, whereas those in the surgery group did not receive PPI after gastrectomy. PPI was not routinely administered before either procedure. One patient in the ESD group, who was *H. pylori* positive, underwent eradication therapy shortly after the procedure, with confirmation of eradication via a urea breath test. Fecal samples were collected from the central portion of the fecal samples with sterile swabs, transferred to containers included in the collection kit, and stored at −20 °C until further analysis.

### 2.2. DNA Extraction and NGS Library Preparation for Sequencing

DNA from each fecal sample was isolated using a DNeasy PowerSoil kit (QIAGEN, Düsseldorf, Germany) following the manufacturer’s protocol. The extracted DNA was quantified using the QIAxpert system (QIAGEN). V3–V4 regions of the 16S rDNA gene was amplified with primers; 16S_V3_F (5′-TCGTCGGCAGCGTCAGATGTGTATAAGAGACAGCCTACGGGNGGCWGCAG-3′) and 16S_V4_R(5′-GTCTCGTGGGCTCGGAGATGTGTATAAGAGACAGGACTACHVGGGTATCTAATCC-3′). Index polymerase chain reaction with sequencing adapters attached to the amplified DNA was performed using the Nextera XT Index kit (Illumina, San Diego, CA, USA). After the quantification, normalization, and pooling of the libraries, each amplicon was sequenced using MiSeq (Illumina).

### 2.3. Taxonomic Assignment and Profiling

Paired-end 16S rRNA gene sequences were analyzed using the Quantitative Insights into Microbial Ecology (QIIME2 v 2021.4, Northern Arizona University, Flagstaff, AZ, USA) software [14]. The amplicon sequences were trimmed using Cutadapt (NBIS, Uppsala, Sweden). Reads were denoised for quality and chimeric reads using the dada2 method manual parameters (trim-left-f 0, trim-left-r 0, trunc-len-f 260, trunc-len-r 200, trunc-q 2, max-ee-f 3, and max-ee-r 3) [15]. Taxonomic classification was performed using a naïve Bayes classifier trained on the V3–V4 region of the SILVA 138 database [16]. Sequences identified as chloroplasts or mitochondria were excluded from analysis.

### 2.4. Statistical Analysis

Read counts were normalized by rarefying the samples to a minimum of 806 reads. Statistical analysis of the differences between the two groups was performed using the Mann-Whitney U test. Alpha diversity was assessed using the observed Chao1, Shannon, and Simpson indices and rarefied to compare species richness and evenness. Principal Coordinate Analysis (PCoA) was performed to evaluate the differential clustering of samples before and after the procedure. Bray–Curtis dissimilarity was used for PCoA, and the *p*-value was assessed using permutational multivariate analysis of variance with distance matrices (PERMANOVA) with 999 permutations. For analysis of categorical variables, Fisher’s exact test was applied to contingency tables. Statistical significance was set at *p* < 0.05. All statistical analyses were performed using R version 4.4.0 (R Foundation for Statistical Computing, Vienna, Austria).

## 3. Results

### 3.1. Baseline Characteristics of the Enrolled Participants

The study included a total of 13 patients (five with ESD and eight with surgery), with an average age of 66.5 ± 10.3 years. The proportions of male patients and those with stage 1A gastric cancer were 61.5% and 77%, respectively. The baseline characteristics of the patients in the ESD and surgery groups are shown in Table 1.

### 3.2. Comparative Analysis of the Microbiomes of the Pre-Treatment Samples in the ESD and Surgery Groups

**Figure 1:**
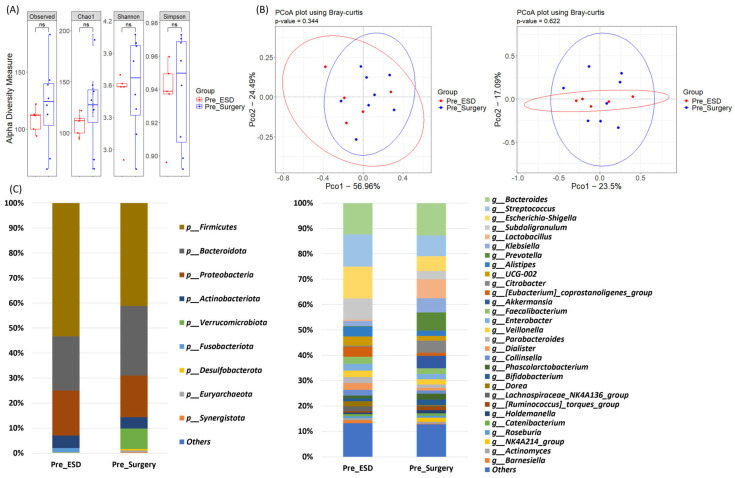
Comparison of alpha diversity, beta diversity, and relative abundance between pre-treatment microbiome in ESD and Surgery groups. (**A**) Alpha diversity measures, including Observed, Chao1, Shannon, and Simpson indices, were compared between the ESD and Surgery groups. (**B**) Principal Coordinate Analysis (PCoA) using Bray-Curtis dissimilarity was performed to examine the beta diversity of the microbiomes at both phylum and genus levels. (**C**) Bar graphs illustrating the microbiome composition at the phylum and genus levels in both groups, showing the relative abundance of major bacterial taxa present before treatment. ns: *p* ≥ 0.05.

We measured the alpha and beta diversities, as well as the microbiome composition in patient samples before the procedure. The alpha diversity of the microbiome was assessed using the observed, Chao1, Shannon, and Simpson indices. No significant differences were found between the ESD and surgery groups before the procedure (*p* > 0.05; Figure 1A), indicating comparable microbial richness and evenness across the patient samples in the two groups. Beta diversity was analyzed using PCoA based on Bray–Curtis dissimilarity. The results showed no significant clustering between the samples from the two groups before the procedure (*p* > 0.05; Figure 1B), suggesting similar microbial community structures. The taxonomic composition of the microbiome was analyzed at the phylum and genus levels. The dominant phyla observed in all samples were Firmicutes, Bacteroidota, and Proteobacteria. However, there were no significant differences in the relative abundance of major taxa at both the phylum and genus levels between the two groups before the procedure (*p* > 0.05; Figure 1C). The microbiome composition at baseline appeared to be similar in both patient groups.

### 3.3. Integrated Analysis of Microbiome Alterations Between the Pre- and Post-Treatment Samples

**Figure 2:**
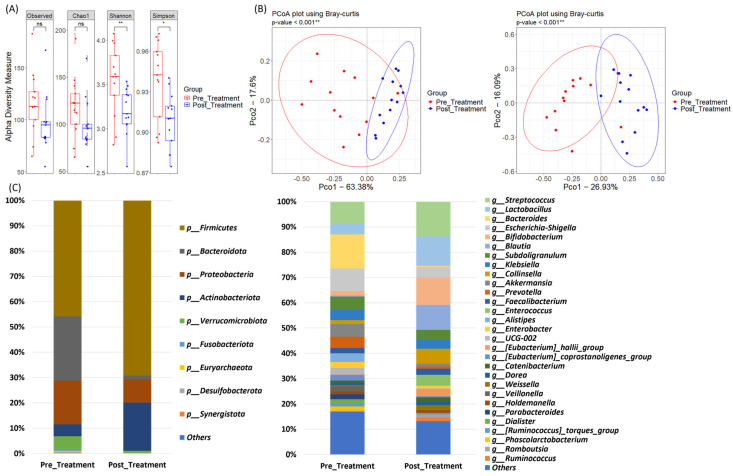
Comparison of alpha diversity, beta diversity, and relative abundance between pre- and post-treatment. (**A**) Alpha diversity measures, including Observed, Chao1, Shannon, and Simpson indices, were compared between the ESD and Surgery groups. (**B**) Principal Coordinate Analysis (PCoA) using Bray-Curtis dissimilarity was performed to examine the beta diversity of the microbiomes at both phylum and genus levels. (**C**) Bar graphs illustrating the microbiome composition at the phylum and genus levels in both groups, showing the relative abundance of major bacterial taxa present before treatment. * *p* < 0.05, ** *p* < 0.01, ns: *p* ≥ 0.05.

A comparison of alpha diversity pre- and post-treatment (including patients with ESD and gastrectomy) revealed a significant reduction in Shannon and Simpson indices post-treatment (*p* < 0.05; Figure 2A). Beta diversity analysis showed significant clustering of samples collected pre- and post-treatment (*p* < 0.05; Figure 2B), suggesting a shift in the microbial community structure following treatment. After treatment, there was a significant increase in the relative abundance of Firmicutes and a decrease in Bacteroidota at the phylum level in all patients (*p* < 0.05; Figure 2C). At the genus level, the relative abundance of Bacteroides significantly decreased (*p* < 0.05; Figure 2C), whereas those of Lactobacillus, Bifidobacterium, and Blautia showed a significant increase (*p*< 0.05; Figure 2C), indicating a notable shift in specific microbial taxa after the procedures.

### 3.4. Microbiome Dynamics in the ESD Group: Pre- and Post-Treatment Samples

**Figure 3:**
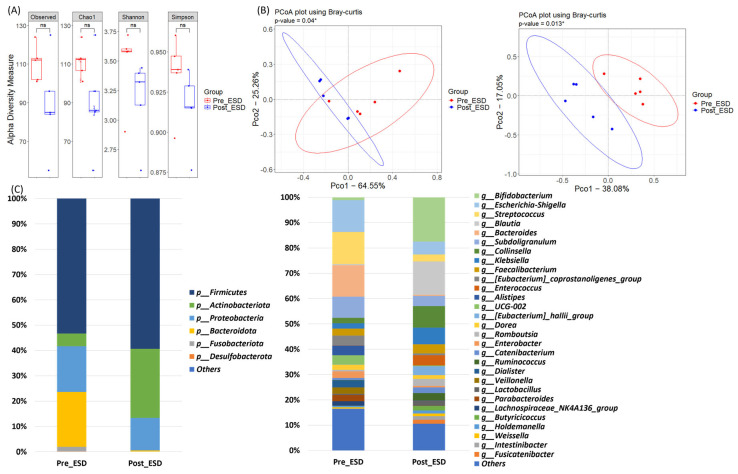
Comparison of alpha diversity, beta diversity, and relative abundance between pre- and post-treatment in ESD group. (**A**) Alpha diversity measures, including Observed, Chao1, Shannon, and Simpson indices, were compared between pre- and post-treatment in ESD group. (**B**) Principal Coordinate Analysis (PCoA) using Bray-Curtis dissimilarity was performed to examine the beta diversity of the microbiomes at both phylum and genus levels. (**C**) Bar graphs illustrating the microbiome composition at the phylum and genus levels in both groups, showing the relative abundance of major bacterial taxa present before treatment. * *p* < 0.05, ns: *p* ≥ 0.05.

We further performed a subgroup analysis by dividing the patients into the ESD and surgery groups to investigate whether there were differences in the microbiome changes before and after treatment according to the treatment modality. In patients who underwent ESD, a decreasing trend in alpha diversity was observed post-treatment; however, this trend was not significant for any index (*p* > 0.05; Figure 3A). Beta diversity analysis revealed significant clustering between the pre- and post-ESD samples (*p* < 0.05; Figure 3B), suggesting a shift in microbial community structure following ESD. However, statistical analysis using the paired Mann-Whitney U test did not reveal any significant differences in the relative abundances of major phyla or genera (*p* > 0.05; Figure 3C).

### 3.5. Microbiome Dynamics in the Surgery Group: Pre- and Post-Treatment Samples

**Figure 4:**
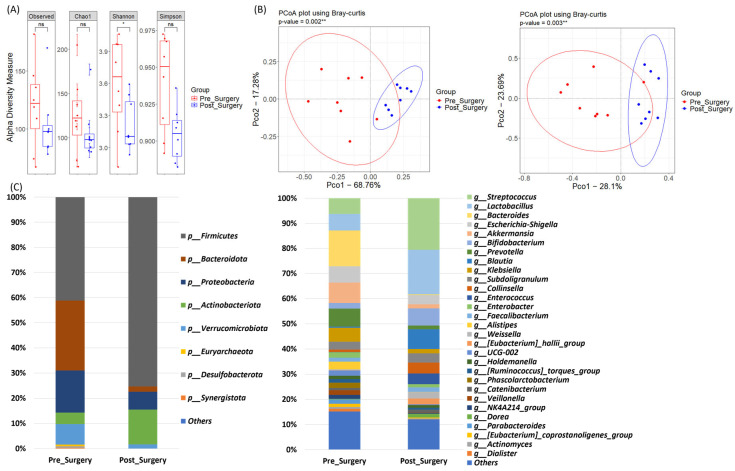
Comparison of alpha diversity, beta diversity, and relative abundance between pre- and post-treatment in Surgery group. (**A**) Alpha diversity measures, including Observed, Chao1, Shannon, and Simpson indices, were compared between pre- and post-treatment in Surgery group. (**B**) Principal Coordinate Analysis (PCoA) using Bray-Curtis dissimilarity was performed to examine the beta diversity of the microbiomes at both phylum and genus levels. (**C**) Bar graphs illustrating the microbiome composition at the phylum and genus levels in both groups, showing the relative abundance of major bacterial taxa present before treatment. * *p* < 0.05, ** *p* < 0.01, ns: *p* ≥ 0.05.

Alpha diversity showed a general decrease after gastrectomy, with a significant difference in the Shannon index (*p* < 0.05; Figure 4A). Beta diversity analysis revealed significant clustering between the pre- and post-gastrectomy samples (*p* < 0.05; Figure 4B), indicating a notable shift in the microbial community structure following surgery. After gastrectomy, there was a significant increase in the relative abundance of Firmicutes and a decrease in that of Bacteroidota at the phylum level (*p* < 0.05; Figure 4C). At the genus level, the relative abundance of Streptococcus and Blautia significantly increased, whereas that of Bacteroides significantly decreased (*p* < 0.05; Figure 4C), reflecting a substantial change in microbiome composition.

## 4. Discussion

Our findings indicate significant differences in both alpha and beta diversities following treatment, revealing distinct patterns of post-treatment microbiome changes between the ESD and surgery groups.

Previous studies examining post-gastrectomy microbiome changes in patients with gastric cancer have consistently reported shifts in microbial composition and diversity, often showing a reduction in microbial diversity alongside shifts in specific taxa [8,17]. Consistent with these findings, we observed a general decrease in alpha diversity in the gastrectomy group and a significant shift in beta diversity in the gastrectomy group. This reduction in microbial diversity is frequently attributed to the decreased stomach acid production after gastrectomy. This shift alters the gut environment, increasing the pH in the stomach and proximal intestines and weakening its protective barrier against bacteria from the oral cavity. Consequently, bacteria that typically do not survive in acidic environments, such as Streptococcus, can persist and thrive in the GI tract [18]. This shift in pH not only promotes the survival of oxygen-tolerant bacteria but also disrupts the balance of obligate anaerobes, leading to decreased overall microbial diversity in the gut. Furthermore, this trend is consistent with reports suggesting that reduced gastric acidity facilitates the persistence of Streptococcus species and other oral commensals in the GI tract [19].

In our gastrectomy cohort, the relative abundances of Streptococcus and Blautia significantly increased, whereas that of Bacteroides decreased postoperatively. These changes align with previous studies, which reported an increase in oxygen-tolerant bacteria, such as Streptococcus, following gastric surgery. The reduced acidity post-surgery creates conditions that favor these bacteria over anaerobic species, like Bacteroides [8]. To expand the potential impact of Bacteroides reduction after gastrectomy, it is essential to understand the significant role of Bacteroides in maintaining gut health through the production of short-chain fatty acids (SCFAs), particularly butyrate, acetate, and propionate. SCFAs, especially butyrate, are well-recognized for their anti-inflammatory properties, which help regulate immune responses, maintain the integrity of the intestinal barrier, and prevent inflammation in the gut [20,21]. A decline in Bacteroides, which are responsible for substantial SCFA production, can reduce the availability of these crucial compounds. This reduction may compromise the anti-inflammatory environment of the colon and promote low-grade inflammation, which could in turn support the development of colorectal neoplasms and inflammatory bowel disease-like symptoms over time [22]. Furthermore, SCFAs play an important role in maintaining the integrity of the intestinal mucosal layer, as butyrate serves as an essential energy source for colonocytes and supports mucosal barrier function [23,24]. When Bacteroides and other SCFA-producing microbes are reduced, the mucosal barrier may weaken, potentially allowing increased gut permeability, which has been associated with conditions, such as colorectal cancer and inflammatory bowel disease [25,26]. The post-gastrectomy environment, with its elevated pH and composition, may exacerbate this effect, creating a cycle of dysbiosis and inflammation that could increase long-term health risks [17]. This observed microbial imbalance after gastrectomy suggests the need for monitoring and possibly modulating the gut microbiome in patients with gastric cancer, especially given the potential implications of dysbiosis on postoperative outcomes and in preventing long-term risks, such as colorectal neoplasms [17,27].

Comparatively, ESD, which is a less invasive procedure that preserves stomach structure and function, appears to induce subtle microbiome changes and has a minimal impact on gastric acid secretion. Unlike gastrectomy, which significantly reduces acid production owing to the partial or complete removal of the stomach, ESD leaves most of the gastric anatomy intact, thus maintaining a more stable pH and acid production environment in the stomach and proximal intestines. The preservation of acid levels likely plays a role in limiting microbiome alterations following ESD. In our study, although the alpha diversity metrics suggested some changes after ESD, these shifts were not statistically significant and appeared notably less pronounced than those observed in the gastrectomy group. This finding suggests that the microbiome changes observed after gastrectomy are partly driven by anatomical and physiological alterations resulting from surgery, rather than by cancer treatment alone [28,29]. Limited studies on ESD have reported minor alterations in microbial composition, but their impact on the gut microbiome remains relatively modest compared to that of more invasive surgeries [30]. The changes in the microbiome in ESD group might be partially due to lifestyle modifications including dietary changes following gastric cancer treatment, for example, which can cause shifts in microbial diversity and composition over time. Additionally, since distinct differences in microbiome composition between gastric cancer tissues and surrounding non-cancerous tissues have been well-documented, the removal of the tumor itself may have contributed to these changes [31]. The tumor microenvironment, including the microbiome, interacts dynamically with the cancer, and its resection could lead to shifts in microbial diversity and composition over time, although its impact is likely modest [32]. However, further studies are needed to clarify the extent and mechanisms of these changes.

The differences in microbial composition between the ESD and gastrectomy groups could also account for variations in postoperative GI symptoms. Patients undergoing gastrectomy often report lower GI symptoms, such as bloating and diarrhea, likely stemming from a combination of mechanical and microbial factors. These symptoms may be linked to structural changes after gastrectomy, including reduced stomach volume, altered bile acid metabolism, and subsequent dysbiosis, where microbial homeostasis is disrupted [33]. The increased prevalence of Streptococcus and Blautia, both of which are associated with GI discomfort and inflammation, could further exacerbate these symptoms [34]. Conversely, the preservation of stomach anatomy in ESD may help mitigate dysbiosis and related clinical symptoms, underscoring the importance of treatment modalities on patient health status.

The design of this study, which exclusively included patients with stage I gastric cancer enhanced the validity of our findings by minimizing confounding effects related to cancer progression. Although the sample size was small, we tried to enhance comparability between groups through careful participant selection and a long-term follow-up approach to better understand sustained microbiome alterations post-treatment. Moreover, by utilizing a gastric cancer cohort rather than healthy controls, our study uniquely isolated the impact of treatment type on the microbiome, rather than the effects of the cancer itself. According to routine clinical practice, patients in the ESD group enrolled in this study typically received PPI therapy for 4–8 weeks post-procedure, while patients in the gastrectomy group were administered short-term antibiotics pre- and post-surgery, with PPI not administered during this period. Since these medications are known to influence the gut microbiome, their effects should be considered alongside the impact of resection itself. Previous studies have demonstrated that short-term PPI use affects the gut microbiome but that it tends to recover to a baseline state approximately 2–4 weeks after discontinuation [35,36,37]. Similarly, it has been reported that gut microbiome restoration occurs within about 1.5 months following antibiotic use and within one year after H. pylori eradication therapy, resembling the pre-treatment state [38,39]. Therefore, the gut microbiome changes observed in our study, which tracked patients for six months to two years, are more likely attributable to the resection treatments themselves rather than to medication effects.

However, some limitations of our study must be acknowledged. This stringent selection process, excluding patients on inhibited medication and including only those with long-term follow-up and paired pre- and post-treatment fecal samples, resulted in a small sample size. The relatively small sample size and single-center design may have limited the generalizability of the results. In addition, changes in dietary habits or different anastomosis methods in the surgery group might also have influenced the study results. Furthermore, the exclusion of patients with advanced cancer limited the scope of our findings. In addition, while this study provides valuable insights into the alterations in gut microbiota following gastric cancer treatments, the long-term consequences of these changes remain unexplored. Therefore, the results from the study should be interpreted with caution. Future studies with extended follow-up periods are essential to assess whether the observed microbiome changes persist, stabilize, or return to baseline over time. Such research could also elucidate the potential impact of these long-term microbiota shifts on patient health, including risks of neoplasms of lower gastrointestinal tract and other complications. In addition, larger multicenter studies including investigation of microbiome-targeted therapies are needed to help mitigate dysbiosis-related symptoms and potentially reduce long-term health risks in patients with gastric cancer.

## 5. Conclusions

This study provided evidence that ESD and gastrectomy lead to distinct changes in the gut microbiome of patients with gastric cancer. By highlighting these treatment-specific microbiome alterations, our findings underscore the importance of understanding how these changes influence patient care and recovery processes following gastric cancer treatment. Further research is needed to explore the clinical implications of these microbiome shifts and determine how they can be utilized to optimize post-treatment strategies for gastric cancer management.

## Figures and Tables

**Table 1:** Baseline characteristics of the enrolled subjects.

Variables	Total (*n* = 13)	ESD Group (*n* = 5)	Surgery Group (*n* = 8)	*p*-Value
Age, year	66.5 ± 10.3	60.6 ± 10	70.1 ± 9.3	0.1064
Sex, male/female	8/5 (61.5%/38.5%)	4/1 (80%/20%)	4/4 (50%/50%)	0.5649
Histology				0.2929
W/D and M/D	8 (61.5%)	5 (100%)	3 (37.5%)	
P/D	2 (15.4%)	0 (0%)	2 (25%)	
PCC	2 (15.4%)	0 (0%)	2 (25%)	
MiNEN	1 (7.7%)	0 (0%)	1 (12.5%)	
Stage				0.2308
IA	10 (76.9%)	5 (100%)	5 (62.5%)	
IB	3 (23.1%)	0 (0%)	3 (37.5%)	
T stage				0.3147
1a	9 (69.2%)	4 (80%)	5 (62.5%)	
1b	1 (7.7%)	1 (20%)	0 (0%)	
2	3 (23.1%)	0 (0%)	3 (37.5%)	
N stage				-
0	13 (100%)	5 (100%)	8 (100%)	
1	0 (0%)	0 (0%)	0 (0%)	
Lymphovascular invasion				0.4872
Absent	11 (84.6%)	5 (100%)	6 (75%)	
Present	2 (15.4%)	0 (0%)	2 (25%)	
Gross type				1
Elevated	2 (15.4%)	1 (20%)	1 (12.5%)	
Flat	3 (23.1%)	1 (20%)	2 (25%)	
Depressed	8 (61.5%)	3 (60%)	5 (62.5%)	
Location				0.4872
Upper third	0 (0%)	0 (0%)	0 (0%)	
Middle third	2 (15.4%)	0 (0%)	2 (25%)	
Lower third	11 (84.6%)	5 (100%)	6 (75%)	
Anastomosis method				-
Billroth I	-	-	2 (25%)	
Billroth II	-	-	1 (12.5%)	
Roux-en-Y	-	-	5 (62.5%)	

Values are expressed as mean ± standard deviation or number (percentage), unless otherwise specified. ESD, endoscopic submucosal dissection; W/D, well-differentiated; M/D, moderately-differentiated; P/D, poorly-differentiated; PCC, poorly cohesive carcinoma; MiNEN, mixed neuroendocrine non-neuroendocrine neoplasm.

## Data Availability

The datasets used and/or analyzed during the current study are available from the corresponding author on reasonable request.
